# Customizing cellular signal processing by synthetic multi-level regulatory circuits

**DOI:** 10.1038/s41467-023-44256-1

**Published:** 2023-12-18

**Authors:** Yuanli Gao, Lei Wang, Baojun Wang

**Affiliations:** 1 College of Chemical and Biological Engineering & ZJU-Hangzhou Global Scientific and Technological Innovation Center, Zhejiang University, Hangzhou, 310058 China; 2 School of Biological Sciences, University of Edinburgh, Edinburgh, EH9 3FF UK; 3 Center of Synthetic Biology and Integrated Bioengineering & School of Engineering, Westlake University, Hangzhou, 310030 China; 4 Research Center for Biological Computation, Zhejiang Lab, Hangzhou, 311100 China

**Keywords:** Synthetic biology, Genetic circuit engineering, Synthetic biology

## Abstract

As synthetic biology permeates society, the signal processing circuits in engineered living systems must be customized to meet practical demands. Towards this mission, novel regulatory mechanisms and genetic circuits with unprecedented complexity have been implemented over the past decade. These regulatory mechanisms, such as transcription and translation control, could be integrated into hybrid circuits termed “multi-level circuits”. The multi-level circuit design will tremendously benefit the current genetic circuit design paradigm, from modifying basic circuit dynamics to facilitating real-world applications, unleashing our capabilities to customize cellular signal processing and address global challenges through synthetic biology.

## Abstract (web summary)

As synthetic biology permeates society, the signal processing circuits in engineered living systems must be customized to meet practical demands. In this review, the authors outline design strategies for the DNA, RNA, and protein-level circuits and the hybrid “multi-level” circuits.

## Introduction

Synthetic biology aims to engineer genetic circuits in living systems for user-defined behavior. These living systems, such as bacteria, yeast, plant, and mammalian cells, have been programmed to produce high-value chemicals and materials, diagnose and treat diseases, monitor environmental contaminants, and improve crop yields^1,2^. Devising such living systems relied on combining the growing understanding of biological processes with the principles and disciplines of electronic engineering. Like electronic circuits, a synthetic genetic circuit comprises three modules: sensor, signal processor, and actuator^3^. The sensor module transduces extracellular signals (inputs) into intracellular signals, which are integrated and computed by the signal processing module. The actuator module converts processed information to desired physiological activities (outputs). As interconnecting circuits wiring the sensor to the actuator, the signal processing circuits are essential for tuning the input-output relationships and achieving complex functions.

**Fig. 1:**
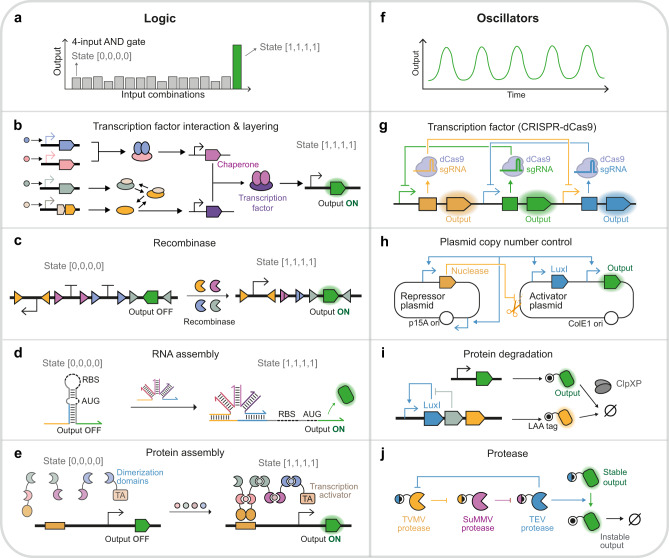
Synthetic logic computation and oscillation. Recent examples of synthetic logic circuits are shown in (**a–e**) and oscillators in (**f–j**). **a** Four-input AND gates only produce high output signals at the state [1, 1, 1, 1] where four input signals are all present. **b** The four-input AND gate based on the transcription factor (TF) interaction and layering^34^. The two-input AND gates relying on the interaction of the transcriptional activator and its cognate chaperone protein are layered into the four-input AND gate. **c** The four-input AND gate based on recombinase-mediated inversion of promoter and coding sequences, and excision of terminators^4,43^. **d** The four-input AND gate based on the assembly of trigger RNAs of the toehold switch^12^. The trigger RNA complex initiates strand displacement and exposes the RBS and start codon to activate translation. **e** The four-input AND gate based on the chemical-induced protein assembly^84^. Four chemical-induced dimerization domains bridge the TF DNA-binding domain and transcription activation (TA) domain. **f** Oscillators produce periodic, oscillating outputs. **g** The oscillator based on the CRISPR-dCas9 system^32^. The upstream sgRNA binds with dCas9 and represses the transcription of the downstream sgRNA. **h** The oscillator based on plasmid copy number control^53^. The activator plasmid encodes a self-activating quorum-sensing LuxI synthase and P_luxI_-driven reporter protein. The repressor plasmid encodes a P_luxI_-driven endonuclease cleaving the activator plasmid and P_luxI_-driven RNA mediating repressor plasmid replication. **i** The oscillator based on the post-translational coupling of genetic circuits^85^. The quorum clock (driving the yellow protein expression) and constitutively expressed green protein are coupled by sharing ClpXP proteases. **j** The oscillator based on protein cleavage and degradation^87^. The upstream protease (e.g., TEV protease) exposes the degron fused to the downstream protease (TVMV protease) and reporter proteins, leading to degradation.

Over the past decade, signal processing circuits have developed significantly in scale and function, generating spatiotemporal output signal patterns in response to different strengths, durations, frequencies, combinations, and temporal order of input signals (Table 1). Boolean logic circuits are the most fundamental and prevalent, processing digital signals with distinct ON and OFF states (Fig. 1a). Combinational logic circuits executing various functions, like addition/subtraction^4,5^, majority^6–9^, encoding/decoding^4,7^, and multiplexing/demultiplexing^7,9,10^, have been constructed from basic logic gates (Buffer, NOT, AND, NAND, NOR, NIMPLY, IMPLY, XOR, and XNOR gates). The most impressive demonstrations are a set of 37 three-input circuits designed by Cello^9^, a 6-input Boolean Logic Look-Up Table^4^, and a 12-input disjunctive normal form ribocomputing circuit^11^. The Boolean logic circuits can be wired in a closed-loop way to devise sequential logic circuits^12^, whose states depend on current input signals and input histories. These circuits and recombinase-based memory circuits are employed to build state machines^13–15^, which stably remain in current states until specific signals are received for irreversible transition to other states.

**Table 1:** Recent examples of cellular signal processing circuits based on different regulatory systems

Circuit		Description	Host	Examples of regulatory systems
Signal generator	Pulse generator	A circuit that generates pulses	*E. coli*	Protease^88^, riboregulator^57^
	Oscillator	A circuit that generates periodic waves	*E. coli*	Transcription factor (TF)^33^, protease^87^, protein degradation^85^, plasmid copy number control^53^
Signal converter	Analog-to-digital converter	A circuit that converts analog inputs into digital outputs based on different thresholds	*E. coli*	Recombinase^103^, TF^6^
	Digital-to-analog converter	A circuit that converts digital inputs into analog outputs	*E. coli*	Recombinase^138^, TF^39^
Signal filter	Band-pass filter	A circuit passes a specific input range but rejects inputs outside the range	*E. coli*, mammalian, yeast	*Input-strength filter:* recombinase^103^, TF dimerization^84^, protease^88^, riboswitch^61^ *Input-frequency filter:* TF cooperative assembly^19^
	Band-stop filter	A circuit rejects a specific input range but passes inputs outside the range	Yeast	*Input-frequency filter:* TF cooperative assembly^19^
	Persistence filter	A circuit that passes inputs of sufficiently long durations	Yeast	TF cooperative assembly^19^
Analog logic	Adder	A circuit that sums two inputs	*E. coli*	TF-based positive feedbacks^16^
	Ratiometer / Division	A circuit that returns the ratio of two inputs	*E. coli*	TF-based positive feedbacks^16^, Protease-based incoherent network^139^
Boolean logic (Two-valued logic)	Logic gates	AND, NAND, NOR, NIMPLY, IMPLY, XNOR, BUFFER, NOT gates	*E. coli*, mammalian, yeast, plant	Toehold switch^11^, protein heterodimers^83,84^, protease^88^, recombinase^4,41,44^, TF^9,23,25,26,109^, miRNA & RNA binding protein^72^, endoRNase^87^
	Adder Subtractor	Adder carries the addition of digital inputs. Subtractor carries the subtraction of digital inputs.	Mammalian	Recombinase (*full adder, full subtractor, half adder-half subtractor*)^4^, CRISPR-dCas9 (*half-adder*^5^*, half-subtractor*^69^)
	Encoder Decoder	Encoder converts multiple input signals to a smaller number of output signals. Decoder converts multiple input signals to a larger number of output signals.	*E. coli*, mammalian	TF (*4-to-2 decoder, 2-to-4 decoder*^7^), recombinase (*2-to-4 decoder*)^4^
	Multiplexer Demultiplexer	Multiplexer propagates one of all input signals to one output signal depending on the select signals. Demultiplexer propagates one input signal to one of all output signals depending on the select signals.	*E. coli*	TF (*2-to-1 multiplexer, 1-to-2 demultiplexer*)^7,10^
	Majority	A circuit produces the value of the majority of the inputs. For example, the circuit returns 1 if most inputs are 1.	*E. coli*, mammalian	TF^6–8^
	Other combinational circuits	a. Disjunctive normal form (DNF) circuit: A circuit that uses OR gate to connect inputs from multiple AND and NOT gates b. Two sets of 37 and 5 three-input logic gates were designed by Cello and functional in *E.coli* and yeast, respectively.	*E. coli*, mammalian, yeast	Toehold switch (*up to 12-input DNF circuit*)^11^, TF^9,23,39^, recombinase^4^
Multi-valued logic	Ternary / three-valued logic	A circuit that produces three output states	*E. coli*, mammalian	Recombinase^103^ (*two outputs*), protein dimerization^84^ (*two outputs*), TF^6^ (*one output*)
Reversible logic	Feynman gate Fredkin gate	Feynman gate is a two-input-two-output logic gate with one-to-one input-output mapping. Fredkin gate is a three-input-three-output logic gate with one-to-one input-output mapping.	*E. coli*	TF^7^
Memory	Bistability	A circuit that switches between two states to produce two outputs with two inputs.	*E. coli*, mammalian, yeast, plant	TF^12^, endoRNase^72^, recombinase^47,140^, protein phosphoregulation^101^
	State machine	A circuit that switches among up to four states, with checkpoint signals	*E. coli*	TF^12^, TF & protein dimerization^13^, Recombinase^14,15^
Artificial Neural networks	Perceptron	A neural network unit that carries the weighted sum of inputs and produces functions of outputs	*E. coli*	Metabolic pathway^132^, TF^6,7^
Genetic controller	Feedback controller	A genetic controller that compares the output levels to a set point (input level), and if these levels are different, the controller will restore the original output level.	*E. coli*, mammalian, yeast	Protein splicing^95^, protein sequestration^116^, asRNA^117–119^
	Feedforward controller	The feedforward controller directly senses the disturbance and offsets its effect to restore the original output level.	*E. coli*, mammalian	miRNA^120^, endoRNase^73^

Analog logic circuits process continuous variable signals and perform arithmetic calculations like addition, division, multiplication, and power law^6,16^. Signal filters like band-pass and band-stop filters are also common devices to process analog signals, passing signals within specific ranges of strengths or frequencies and filtering those beyond that range. The analog and digital signal processing was further integrated for neuron-like computation^6,7^, which linearly combines weighted input signals, and nonlinearly computes them to produce digital outputs. The pioneering works in neuron-like computation enable un-classical logic computations (e.g., multi-valued logic^6^ and reversible logic^7^) and signal converters^6^. The signal converters with different thresholds can achieve the transition between digital and analog signals (analog-to-digital conversion and vice versa). Moreover, the output dynamics can be tuned by precisely devising the topologies of genetic networks, resulting in pulses and oscillations.

The implementation of signal processing circuits follows a bottom-up strategy analogous to their electronic counterparts; that is, artificial circuits are assembled from a set of composable biological parts in a “plug-and-play” manner. The regulatory parts usually comprise two elements, one regulating the other for inducible and tunable functionality, bestowing the circuits with distinct functions and dynamics. This review summarizes the explosive growth of regulatory parts and their achievements in synthetic signal processing, emphasizing the novel regulatory modalities reported in recent years. We classify these parts according to the levels of central dogma: DNA, RNA, and protein, and discuss their advantages and challenges (Table 2). Furthermore, we explore the integration of different regulatory parts into a hybrid genetic circuit, which we term “multi-level regulation”, a previously neglected circuit design strategy. We discuss several aspects in which this multi-level regulation can contribute to current signal processing paradigms, from altering basic response profiles to expediting real-world applications. Finally, we discuss the challenges and opportunities for customizing these signal processing circuits.

**Table 2:** The advantages and challenges of different regulatory systems

	Advantages	Challenges	Niches in multi-level circuit
Transcription factors	• The TF systems are extensively studied, and design automation tools^9,23,35^ and well-established libraries are available. • The modular design allows concatenating different modules into complex topologies. • The programmable TFs can target endogenous pathways.	• The transcriptional circuits show slow kinetics, especially in layered design. • The transcriptional circuits suffer from retroactivity, burden, and large genetic footprints. • The layered design is sometimes overcomplicated for simple logic gates.	• Sensor • Genetic “wire” to connect different modules and tune signal levels • Genetic controller • Layered logic • Dynamic control
Recombinase	• The low basal level produces tight OFF states and digital response. • Recombinase-based circuits are genetically compact. • Irreversible recombination is suitable for memory and cascade. • Design automation tools^15,42,137^ and well-established libraries are available.	• The use of repetitive recognition sites may decrease recombination specificity and genetic stability^15^. • Some recombinase recognition sites show cryptic promoter or terminator activities^15^. • The recombinase-based circuits show slow kinetics.	• Digitizer • Recording and memory • State machine (synthetic differentiation) • Single-layer DNA-level logic
Plasmid copy number (PCN) control	• Changing PCN can simultaneously alter the expression of all genes encoded in the plasmid. • Reduced PCN is coupled with cell viability under antibiotic selection.	• The kinetics is slow, constrained by cell division. • The number of plasmids used in the same cells is restricted by plasmid incompatibility.	• Global gene expression control • Cell viability control (kill switch)
Riboregulator	• The RNA base-pairing is programmable and predictable, affording in silico design, prediction, and large orthogonal libraries. • The de novo*-*designed assembly of multiple RNA strands is suitable for multi-input signal processing. • The RNA circuits have small genetic footprints, fast kinetics, and low metabolic load. • Riboregulators can respond to endogenous RNAs.	• The riboregulators usually require high expression levels of trans-acting RNAs. • Promiscuous interactions with host transcriptomes potentially affect bacterial growth^104^. • Promiscuous interactions with surrounding genetic contexts (e.g., insulator^141^) affect modularity. • Some riboregulators change the protein sequences of genes of interest.	• Endogenous RNA sensor • Post-transcriptional multi-input logic • Dynamic control
Riboswitch & Ribozyme	• Riboswitches and ribozymes are functional across different host organisms. • Ribozymes are amenable to aptamer insertion, allowing external regulation. • Computational tools to design riboswitches are available.	• Limited parts are available for RNA aptamers, and identifying new aptamers tends to be hard. • The dynamic ranges of riboswitches are usually low.	• Sensor • Dynamic control
RNA inference (miRNA & asRNA) & RNA binding protein	• The circuits encoding these modalities could be delivered by RNA and regulated by external inducers and endogenous miRNA biomarkers • The RNA-binding proteins could amplify post-transcriptional signals and interface with protein-level regulation	• The function of miRNAs and asRNAs involves endogenous machinery, potentially causing queuing effect and burden. • The gene activation mechanisms are few; in most cases the gene expression can only be repressed. • The lifetime of RNA-delivered circuits is relatively short.	• Endogenous miRNA sensor (cell classifier) • Genetic controller • Post-transcriptional logic based on NOT gates • Transient gene expression for a short period
Programmable RNA-targeting system	• The sequence-specificity is high and the ADAR-based systems can distinguish dinucleotide variants^76^. • The RCas system can deliver diverse effector proteins to execute functions more than activation and repression.	• The systems are not fully programmable, as the ADAR system recognizes specific codons in target RNA. • The ADAR-based translational control can only produce protein signals and produce peptides that may vary in immunogenicity^77^. • The large size and bacterial origin of the dCas protein hinder its application in eukaryotic systems^80^.	• Endogenous RNA sensor (cell classifier) and editor
Protein regulators	• The protein circuits operate at fast kinetics^100^ • The protein interactions are functional across different cellular compartments and various host contexts • The protein circuits can easily interface with endogenous protein processes^101^ • The protein regulators can function in RNA-delivered circuits	• Limited parts are available, restricting the scale-up of protein circuits. • Overloading protein degradation machinery causes queuing effect^87^. • Protein proteolysis costs relatively high levels of energy like ATP^87^. • Inserting protein interaction domains to target effectors may disrupt the protein functionality and needs carefully selecting insertion locations.	• Signal transmitter between endogenous signaling pathway and synthetic circuit • Genetic controller • Post-translational logic • Dynamic control

## DNA-level signal processing

### Transcription factor (TF)

Transcription factors are DNA-binding proteins that interpret DNA-level information into RNAs by manipulating the transcription activities. Transcription activators recruit transcription machinery to specific promoter sequences. In contrast, transcription repressors compromise transcriptional activities by blocking the transcription initiation or elongation to invert the signals. Recent progress in understanding and designing the TF-promoter and TF-inducer interactions has enabled complex transcriptional programs. The TF-based circuit behavior is rendered more designable through protein interactions, as reviewed in “protein-level regulation” and “multi-level regulation”.

The TF DNA-binding domains (DBD) and promoter sequences have been diversified to establish orthogonal libraries. DNA-binding proteins such as CRISPR-dCas^17^, zinc-finger proteins (ZFPs)^18^, and transcription activator-like effectors (TALEs)^8^ utilize programmable sgRNAs or rearrangeable arrays of protein domains to target any user-defined DNA sequences, allowing them to be developed into orthogonal transcription repressors or activators via conjugation with different effector domains^17,19,20^. By contrast, a set of bacterial TFs, such as helix-turn-helix (HTH) TFs and sigma factors, can only recognize specific operator sequences and are less programmable. Therefore, to exert orthogonal control over different target genes, libraries of bacterial TFs with their cognate operators have been mined from the genomes of miscellaneous organisms, screened, and evolved for better functionality and orthogonality. The library of extracytoplasmic function (ECF) sigma factors is one of the largest, containing 52 functional and 20 orthogonal ECF sigma factors^21^. Likewise, 73 TetR homologs were screened to identify 20 viable and 16 orthogonal repressors^22^. These TetR homologs, as well as other HTH repressors (e.g., LacI homologs and bacteriophage repressors), have been transplanted to eukaryotes (yeast^23^, mammalian^24^ and plant cells^25^) as DNA-binding domains for developing synthetic TFs (sTFs), where they were repurposed into activators via fusion of eukaryotic activation domains.

The transcription activator-based BUFFER gates and repressor-based NOT gates lay the foundations for transcriptional logic^26^. The dose-response performance of these gates could be described by several parameters in the Hill function, namely maximal/basal level (ON/OFF state levels), Hill constant (transition threshold), and Hill coefficient (ultrasensitivity). Notably, ultrasensitivity, or non-linearity, promotes the implementation of layered digital logic circuits, memory, or dynamic circuit behaviors. Many strategies have been proposed to diversify the TF response profiles, such as promoter engineering^27^ and DNA sponge titration^28^, and have seen intensive applications in tuning biosensor behaviors, as reviewed in^29^.

**Fig. 2:**
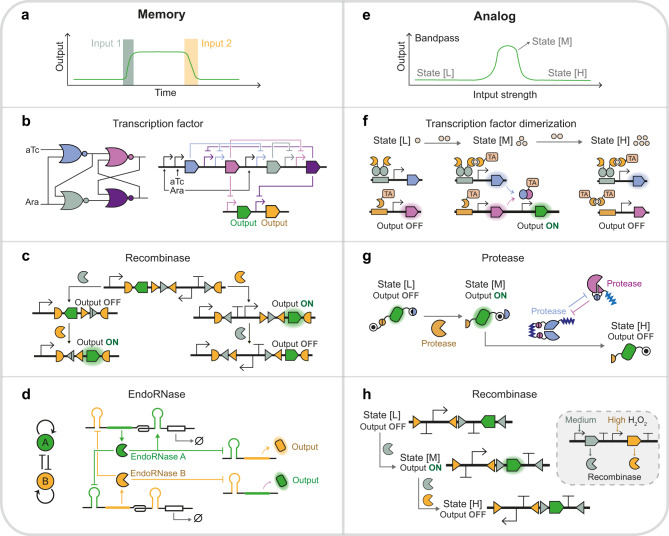
Synthetic memory and analog signal processing. Recent examples of synthetic memory circuits are shown in (**a–d**) and band-pass circuits in (**e–h**). **a** Memory circuits remain in a state until receiving a specific input signal. **b** D latch based on cross-connected NOR gates^12^. **c** State machine based on recombinase with intervened recognition sites^14^. The Bxb1 recombinase (orange) recognizes two orthogonal pairs of recognition sites (triangles and half-ovals). **d** Bistable switch based on endoRNase^72^. Left, the regulatory network motif of genes encoding two endoRNases. The endoRNase A (green) autoactivates its expression and inhibits endoRNase B (orange), and vice versa. Right, the endoRNase autoregulates its translation by cleaving off the degradation signal (white box) and represses the other endoRNase’s expression by cleaving its 5ʹ UTR. **e** Band-pass circuit produces low output levels in response to the low (state [L]) and high (state [L]) range of input strengths but generates high output levels in response to the medium range (state [M]). **f** Band-pass circuit based on transcription factor dimerization^84^. Only at state [M] are the protein monomers (blue and purple) expressed and assembled into synthetic TFs to activate output gene expression. TA, transcription activation domain. **g** Band-pass circuit based on protein cleavage and degradation^88^. At state [M], the TEV protease (orange) is expressed to cleave the degron off the reporter. At state [H], the TVMV protease (blue) exposes the other degron to reduce the reporter abundance. The HCV protease (purple) regulates TVMV protease activity and tunes the band-pass response. **h** Band-pass circuit based on recombinase^103^. At state [M], the expression of Bxb1 recombinase (gray) inverts the coding sequence of the reporter to activate its expression. At state [H], the phiC31 recombinase (orange) is expressed to invert the promoter to repress reporter expression.

Complex transcription programs were rendered possible by layering simple logic gates. The BUFFER gates were wired consecutively into cascades for signal amplification^25,30^ and time-delayed response^31^. The NOT gates, when connected into various network topologies, could generate the most classical circuit behaviors like bistability and oscillation^32,33^ (Fig. 1g). The AND gates were also layered to process more input signals, giving rise to the first four-input AND gate^34^ (Fig. 1a-b). The NOT and NOR gates were wired to implement 16 two-input logic gates^8,9^ and combinatorial circuits. A milestone in layering these gates was Cello^9^, a genetic circuit design automation software that concatenates TetR homolog-based NOT/NOR gates into tailored circuits with user-defined truth tables. The designed circuits could then be mapped into DNAs from *E. coli*^9^, yeast^22^, and gut resident species *Bacteroides*^35^. Moreover, the Cello software allows signal matching of interconnected feedback loops, exploited to design sequential logic circuits for cellular checkpoint control^12^ (Fig. 2b).

Besides the layered design, the single-layer integration of multiple transcriptional signals was also achieved by designing hybrid promoters for competitive or synergistic TF binding. On the one hand, the competitive binding was engineered by rendering the different operators adjacent or overlapping. The competitive binding between the activator and repressor usually resulted in deactivation^25^, whereas that between two dCas9-sgRNA complexes caused derepression of the target promoter^36^. Interestingly, the competitive binding could be directional, as the polarized TF displacement was observed that the upstream-bound TALE could displace the downstream-bound TFs (TALEs, dCas9s, and ZFPs) from DNA but not vice versa^34^. On the other hand, the synergistic binding of different activators to a hybrid promoter could result in AND^20^ or OR^25^ behavior, depending on promoter architectures. Remarkably, Donahue et al.^20^ realized a single-promoter three-input AND gate by designing a hybrid promoter containing the operators of three zinc finger activators. Likewise, a single-promoter three-input NOR gate was created by TALE repressors^8^.

The inducer-binding domains allow the allosteric TFs (aTFs) activities to be regulated by small molecules (inducers) or other signals, providing knobs for producing graded signals and executing the analog computing paradigm. The operator and inducer specificity of aTFs can be improved or altered by directed evolution^37,38^ and rational engineering^39^ for induced activation or repression. Recently synthetic aTFs regulated by multiple ligands have been engineered via combative or cooperative ligand binding. For example, a class of GalS-derived aTFs interacts with two ligands, D-fucose and IPTG, in antagonistic manners where adding one inducer migrates the effect of the other one^40^. The cooperative binding was achieved by tethering the allosteric subunits from different repressors. The resulting chimeric repressors only bound with target promoters in the presence of both inducers, producing NAND logic behavior^38^.

### Recombinase

Recombinase mediates site-specific inversion or excision of DNA sequences, changing the orientation or presence of regulatory elements (e.g., terminators and promoters) between the recognition sites—the DNA rearrangement results in the stable memory of the switch between distinct states in all recombinase-based circuits. Different configurations of regulatory elements and recognition sites have been implemented for constructing all two-input logic gates^4,41^ and combinatorial logic circuits in single-layer architectures. With a repertoire of orthogonal recombinases and heterospecific recognition sites, over 100 distinct functional logic circuits have been created, including four-input AND/NAND gates^42,43^ (Fig. 1c), six-input AND gate, and Boolean Logic Look-Up Table^4^. Moreover, as the DNA arrangement reactions are unidirectional and irreversible, the recombinases are suitable for long-term memory of transient signals^44,45^. Consequently, synthetic state machines were built to alter gene expression according to the temporal order of up to three input signals (Fig. 2c), using interleaved orthogonal recognition sites^14,15^ or positioning the recombinases between their cognate recognition sites^46^. By contrast, the recombination directionality factor can reverse the directionality of DNA arrangement catalyzed by its cognate recombinase, enabling a reversible memory switch^47^ in plant cells.

### Copy number

Plasmids are common platforms for exogenous gene expression. Therefore, altering plasmid copy numbers (PCNs) exerts global control over the genetic circuits encoded in specific plasmid vectors, providing a powerful avenue for rapid prototyping and optimizing these circuits. Recently two strategies for inducible and tunable PCNs have been described. The first one is manipulating the plasmid replication mechanism. For example, the transcription rates of priming RNA (RNAp) and inhibitory RNA (RNAi) were diversified by inducible control and mutagenesis to tune the PCNs of ColE1-derived plasmids^48^. The replication of other vectors like pSC101^49^, mini-F^50^, and R6K^51^ origin plasmids relies on protein elements that have been inducibly expressed to enact tunable PCN control. The second strategy is targeted plasmid degradation by nucleases to reduce the PCNs^52^. Baumgart et al.^53^ combined these two strategies to design a PCN oscillator comprising the activator plasmid, harboring the quorum-sensing LuxI synthase and nuclease-recognition site, and the repressor plasmid encoding the P_luxI_-driven nucleases and RNAp (Fig. 1h). The induction of nucleases degraded the activator plasmids to reduce the PCNs and LuxI expression, which in turn affected the nuclease expression and RNAp-driven replication of the repression plasmids, eliciting robust oscillations.

## RNA-level signal processing

### Riboregulator

Riboregulators manipulate gene expression through RNA interaction triggered conformation change. A typical riboregulator comprises a switch RNA regulating target gene expression in *cis* and a *trans*-acting RNA that binds with switch RNA to modulate its conformation and activity. Taking advantage of the programmable nature of RNA molecules and simple base-pairing mechanism, the riboregulators can be de novo designed, resulting in large libraries of biological parts with wide dynamic ranges and low cross-talk levels for multiplexed control of gene expression. The most notable riboregulator is the toehold switch^54^, a translational activator in which the toehold sequence initiates strand-displacement reactions to expose sequestered start codons upon the binding of trans-acting RNAs. The toehold-mediated mechanism has been adapted to de novo design eukaryotic translational activators^55^, bacterial translational repressors^56^, transcriptional activators (STAR)^57^, and gRNA regulators^58^. Other riboregulators leverage loop-linear interactions^59^ and three-way junctions (3WJs)^56^ to transform RNA inputs into protein outputs. The riboregulators have been integrated into sophisticated ribocomputing circuits by designing RNA self-assembly and co-localization^11,56,57^. For example, a four-input AND gate was created by assembling four RNA strands into a trigger complex to activate the toehold switch (Fig. 1d), and a six-input OR gate was built by concatenating six switch modules^11^. Similar ribocomputing architecture can implement multi-input NAND and NOR logic^56^ and disjunctive normal form computation with up to 12 inputs^11^.

### Riboswitch and ribozyme

Riboswitches exploit RNA aptamers to sense diverse signals and trigger conformation changes to *cis*-regulate the transcriptional or translational activities. The riboswitches could be tandemly arranged to assimilate multiple input signals^60^ or perform analog functions like band-pass filtering^61^. Another class of *cis*-acting regulators, ribozymes, catalyze chemical reactions to modify target RNAs. The ribozyme activities can be controlled via aptamer-mediated allosteric modulation^62,63^, antisense-triggered steric-blocking^64^, or split ribozyme-based *trans*-regulation^65^. The self-cleaving ribozymes, such as the hammerhead ribozyme (HHR) and twister ribozyme, inhibit target mRNA expression via cleavage at 3’ untranslated region (3ʹ UTR)^63,64^ to remove the poly(A) tails in eukaryotic systems and activates target mRNA translation by cleavage at 5’ UTR to expose the sequestered RBS^63,66^ in *E. coli*. Following the similar “sequestration-until-cleavage” design, allosteric HHRs can also release the trans-acting RNAs^62^ to regulate the downstream riboregulator activation. This RNA-level signal transduction cascade exhibits a fast dynamic response upon induction, reaching the steady state in 26 min. Another interesting class of ribozymes is the group I intron, catalyzing RNA splicing reactions, in which the intron splices itself off the precursor RNA and ligates flanking exons. Recently Gambill et al.^65^ grafted the split introns into bacterial 5ʹ UTR to separate the RBS and coding sequences (CDSs) into two RNA fragments, which can be rejoined via trans-splicing reactions triggered by the complex of split introns with input RNA. Such ribozyme-based regulation bypasses host translational mechanisms, viable in different bacterial strains and eukaryotic systems^65,67^, but still suffers from moderate dynamic ranges and small part numbers.

### RNA-binding protein

Besides RNA interactions, the RNA-binding proteins (RBPs) also exert control over numerous RNA processes. For example, proteins binding to RNA aptamers at eukaryotic mRNAs’ UTR^68,69^ and intron regions^70^ modulate their translation and splicing activities. A remarkable work in expanding available sets of orthogonal protein-RNA interactions is the verification of 13 orthogonal Cas proteins with their cognate gRNA motifs^69^. Furthermore, RBPs participate in altering RNA turnover, mediated by antisense RNA (asRNA), microRNA (miRNA), and endoribonuclease (endoRNase). In the asRNA system, the Hfq protein acts as an RNA chaperone to facilitate the binding between target RNA and asRNA^71^, repressing bacterial RNA function and causing degradation. Similarly, miRNA-target RNA hybridization recruits protein complexes for gene silence in mammalian systems. The endoRNases execute site-specific RNA cleavage. Recently DiAndreth et al.^72^ engineered a class of CRISPR-specific endoRNases into RNA-level repressors and activators in mammalian cells. Interestingly, the activation function was evoked via cleaving off an RNA degradation signal sequence, similar to the protease-degron interaction (see below section of protein-level signal processing). This dual-function endoRNase system permits 16 two-input logic operations and circuit topologies like feedforward^73^, feedback loop, and bistable switch^72^ (Fig. 2d). In contrast, the RNA lifetime can be elongated by attenuating the effect of RNases, like fusing stabilizing RNA elements^74^ or sequestering the endoRNase cleavage site^75^.

### Programmable RNA targeting systems

Another magnificent progress is the establishment of versatile, programmable platforms by repurposing the RNA editing and RNA-targeting CRISPR-Cas (RCas) system. Recently RNA sensing systems^76,77^ harnessing RNA-editing by adenosine deaminases acting on RNA (ADAR) have been reported. These systems rely on hybridizing sensor RNA and target RNA to trigger ADAR-catalyzed A-to-I conversion, which transforms a stop codon UAG to UIG in sensor RNAs. The UIG is translated by the ribosome as UGG tryptophan codon, allowing the translation of downstream CDSs. Altering configurations of UAG codons and downstream CDSs conferred these editing-based riboregulators with the capability to execute RNA-responsive AND, OR logic, memory^76^, and positive feedback^77^. The RNA-editing functions have also been realized by fusing ADAR to dCas13 protein in RCas system^78^. The RCas system could deliver other effector proteins to target RNAs for degradation, translation modulation^79^ and alternative splicing^78^. Inspired by the RCas system, Rauch et al.^80^ proposed CIRTS, a minimal RNA targeting system consisting of gRNAs and human protein parts with similar functions but smaller circuit sizes.

## Protein-level signal processing

### Protein binding

Protein binding-based regulation is attained by allosteric modulation or effector colocalization. A milestone for the former protein switch is de novo latching orthogonal cage–key proteins (LOCKR)^81^, where a key protein binds with the cage protein to displace and release the latch domain from inhibition. LOCKR-induced degradation is engineered by embedding degron, a protein-degrading signal sequence, into the latch domain and successfully incorporated into feedback control of signaling pathways^82^. The latter design, effector colocalization, can be represented by the cooperatively inducible protein heterodimer (CIPHR) system^83^, in which the effector proteins are fused to monomers of de novo-designed heterodimers (DHD). Thus, the cognate and competitive binding of DHDs can regulate the colocalization and disassociation of effectors. Using the DNA-binding domain and activation domain of transcription factor (TF) as effectors, the protein interactions are converted into transcriptional signals for executing decision-making functions. Furthermore, the inducible protein interactions can modulate the colocalization of effectors by environmental stimuli (e.g., chemicals^84^, temperature, and light^43^). A mammalian band-pass circuit^84^ was built using the same chemical-inducible dimerization (CID) domains to regulate two different TFs, one activating gene expression upon CID while the other one opposite (Fig. 2f). Only at a medium range of inducers the two TFs can both trigger downstream gene expression, producing the output signals. In the same work, Bertschi et al. constructed six four-input logic circuits and a five-input AND gate by serially arranging orthogonal CID domains to bridge TF DNA-binding domains and activation domains (Fig. 1e).

### Proteolysis

Selective proteolysis offers another powerful tool for post-translational signal processing by modifying protein abundance. Targeted protein degradation utilizes the terminal fusion of degron to direct target protein to endogenous or synthetic degradation machinery. The endogenous degradation machinery like ClpXP proteases is limited and will be overloaded when shared by circuits, resulting in queuing effect and coupling of circuit behaviors. This post-translational coupling mechanism was utilized to confer the oscillation function on a constitutively expressed protein by linking it to a quorum clock^85^ (Fig. 1i). In contrast, synthetic degradation machinery like mf-Lon proteases can be exogenously expressed for inducible and tunable protein degradation, and integrated into genetic circuits like toggle switch^86^. Moreover, targeted protein cleavage relies on site-specific proteases to cleave target proteins at specific recognition sites. These two mechanisms, degradation and cleavage, are coupled for controllable protein degradation where the protease cleavage is designed to reveal or remove degrons, to degrade or stabilize target proteins^87,88^. Using a downstream protease as the target protein controlled by the upstream protease, three orthogonal proteases were layered in a loop to construct a protein-level osscilator^87^ (Fig. 1j). This cleavage-degradation scheme is further extended to the CHOMP (circuits of hacked orthogonal modular proteases)^88^ system by incorporating protein dimerization. In this system, the split protease subunits are fused to dimerization domains which reconstitute active protease until being cleaved off by the upstream proteases. Alternatively, the activation of split protease can be implemented by removing an autoinhibitory peptide from the dimerization domain^89^. These systems permit temporal, digital, and analog signal processing, as demonstrated by the pulse generator, two-input logic gates, and band-pass filter (Fig. 2g).

### Protein splicing

Intein-mediated protein-splicing regulates protein activities via peptide ligation. During protein splicing, inteins excise themselves from precursor proteins and covalently join the flanking protein segments (exteins). Split inteins, when expressed as two separate peptides containing one intein half fused to one extein half, can spontaneously self-associate for protein trans-splicing. Split inteins thus ligate protein subunits and reconstitute split effectors for carrying out AND logic functions. Our group has established effective pipelines for screening viable split inteins and split sites in fluorescent reporters and transcription factors^90,91^. We identified 15 functional split inteins with minimal cross-talk and exploited them to build orthogonal NAND and AND gates^90,91^, incorporated into a three-input-three-output combinatorial circuit. Split inteins were also grafted into recombinases to tune their switch efficiency^92^. Multiple orthogonal split inteins could separate a single protein into several segments and rejoin them for multi-input signal processing. For instance, Jillette et al.^93^ implanted five orthogonal split inteins into a marker protein for the simultaneous selection of six transgenic vectors. Besides ligation, the exchange and removal of protein segments are also possible^94,95^. In a recent example, Anastassov and colleagues^95^ designed the intein-splicing reaction to remove the activation domains from TFs to transform a transcriptional activator (precursor protein) into a repressor (spliced product).

### Protein phosphoregulation

Protein phosphoregulation performs rapid and reversible signal processing via phosphorylation and de-phosphorylation modifications catalyzed by kinase and phosphatase. Currently, most related studies modify and rewire endogenous phosphorylation pathways^96,97^ like two-component systems (TCSs) and mitogen-activated protein kinases (MAPK) pathways, with little success in constructing synthetic, orthogonal phosphorylation cascades. To address this challenge, McClune et al.^98^ screened around 5 × 10^8^ variants of histidine kinases (HKs) and their cognate effectors from bacterial TCSs and identified up to nine orthogonal pathways in *E. coli*. The bacterial HKs and effectors were also repurposed and transplanted into mammalian systems for orthogonal signal transduction^99,100^. In parallel, Mishra et al.^101^ designed synthetic signaling pathways in yeast based on chimeric protein fusions comprising ready-binder (RB), phosphor-binder (PB), and effector. The upstream PB domain senses phosphorylation signals and binds with the RB domain of the downstream protein, colocalizing the upstream effector (kinase/phosphatase) with the downstream PB domain to transmit the phosphorylation signals. This synthetic scheme, abbreviated PRIME, is extended to logic NOT and OR gates and combined with endogenous MAPK pathway to create a toggle switch that transits states in 2 min responding to 30-second input pulses.

## Multi-level signal processing

With a wealth of regulatory tools at DNA, RNA, and protein levels, it is enticing to couple them into multi-level hybrid circuits, as ubiquitous in natural regulatory networks. How does the interplay of different regulatory mechanisms contribute to current signal-processing paradigms? Here we devote a section to the current state-of-the-art and discussion of the benefits of synthetic multi-level signal processing.

### Multi-level regulation alters dose responses

Multi-level regulation provides more tuning knobs for precisely adjusting the dose-response curves. Genetic circuits with designable response profiles form the foundation of digital and analog computing paradigms and empower the conversion between digital and analog signals for mixed-signal processing and neural-like computing. Moreover, these circuits can be applied to optimize biosensors’ detection limits and fold changes in real-world applications.

**Fig. 3:**
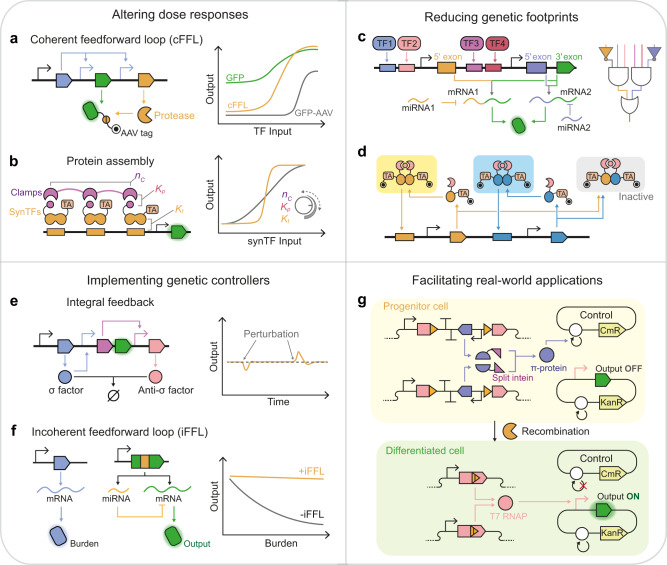
Examples of multi-level regulatory circuits. **a** Coherent feedforward loop (cFFL) circuit based on proteolysis (left) increases the circuit’s dynamic range (right)^30^. **b** Cooperative multipartite protein assembly of the clamp proteins and synthetic TFs (left) digitalizes the circuit’s response (right)^19^. The circuits’ ultrasensitivity can be tuned by altering the number of repeated PDZ domains (n_c_), PDZ-ligand (*K*_*p*_), and synTF-DNA (*K*_*t*_) interaction affinities. TA, transcription activation domain. **c** Six-input disjunctive normal form (DNF)-like circuit comprises the AND, NOT, and OR logic gates based on transcription factor, miRNA, and alternative splicing^108^. TF, transcription factor. **d** Synthetic multistability circuit is built by incorporating transcriptional autoregulation and protein dimerization^13^. The TF homodimers (yellow and blue rectangles) can activate the transcription, whereas the heterodimer (gray rectangle) and monomer cannot. TA, transcription activation domain. **e** Integral feedback controller for robust perfect adaptation^116^ (right) is based on protein sequestration (left). The sigma factor (blue) activates the expression of the reporter (green) and another TF (purple), driving the anti-sigma factor expression to sequester sigma factor activity. **f** Migration of gene expression burden (right) by miRNA-based incoherent feedforward loop (iFFL) (left)^120^. The blue protein expression is used to impose the burden on cellular resources. The miRNAs are transcribed simultaneously with mRNAs to inhibit their translation. **g** Terminal differentiation circuit separates target gene expression and cell viability^51^. In the progenitor cell, the intein-split *π*-protein halves reconstitute functional *π* protein (blue) to maintain the replication of the control plasmid. In the differentiated cell, the expression cassettes of *π* protein are excised by recombinase, resulting in the loss of control plasmid and chloramphenicol resistance. The excision also reconstitutes the coding sequences of T7 RNA polymerase to activate target gene expression. T7 RNAP, T7 RNA polymerase.

One multi-level configuration utilizes the inhibitory RNA and protein interactions, like degradation and protein sequestration, to reduce the basal level and increase the ultrasensitivity of a transcriptional circuit. For instance, the anti-sigma^21^ and exsD^102^ were introduced into ECF sigma-factor and exsA-based BUFFER gates for protein sequestration, upshifting the Hill coefficients of these circuits. These inhibitory interactions were further incorporated into positive feedbacks^102^ or coherent feedforward loops (cFFLs)^30^ for more digital response with greater dynamic ranges. In a seminal cFFL design, the input inducer activates the expression of target proteins and proteases, which cleaves degradation tags off the target proteins to rescue them from protein degradation^30^ (Fig. 3a).

An alternative configuration to tune responses adopts the sequential arrangement of transcriptional control and other regulatory systems. Rubes et al.^103^ connected the H_2_O_2_-responsive TF and recombinase-controlled circuits into genetic comparators with digitalized dose-response curves of H_2_O_2_, whose threshold and transition bands could be shifted by diversifying the RBSs and promoters of recombinases, and further incorporated them into band-pass filters (Fig. 2h). Following a similar strategy, Greco et al.^104^ designed multi-level controllers where a TF drives the switch RNA and trans-acting RNA expression of different riboregulator systems, including toehold switch, STAR, and dual control riboregulator. The integration of RNA-level control altered the basal level, fold change, and ultrasensitivity of transcriptional circuits, depending on the mechanisms of riboregulators. In a separate study, we observed that inserting split intein into TF-regulated proteins significantly decreased the basal activities from upstream transcription circuits^91^. Moreover, cooperative protein binding also reshapes circuits’ response profiles, especially ultrasensitivity, for complex dynamic regulation^105^. Recently Caleb et al.^19^ designed a clamp protein scaffold containing repeated PDZ domains for multivalent assembly with zinc-finger protein TFs (Fig. 3b). The clamp/TF/DNA assembly configuration could be programmed to adjust the transition threshold and Hill coefficient for achieving memory, persistence filtering, and temporal decoding of input pulse in yeast.

### Multi-level regulation tunes time-dependent dynamics

The interplay of regulatory mechanisms operating at distinct timescales permits dynamical control of time-dependent circuit response. One timescale separation configuration (“Fast-Slow”) tandemly layers the fast module and slow module to tune the circuit’s response to time-varying inputs. Gordley et al.^97^ found that linking rapid phosphoregulation with slow transcriptional regulation sensitized the circuit to short input pulses (input duration around 7 min for activating 50% cell population). The transition dynamics and steady-state properties can be independently modified by tuning the fast and slow modules. The fast phosphotransfer cascade was also harnessed to bridge slow transcriptional circuits (“Slow-Fast-Slow”) to implement a load driver device^106^, buffering the connected circuits from retroactive effects: the load-induced time delay and output decrease. Another circuit configuration (“Fast/Slow”) manipulates the expression of the same gene by two parallel mechanisms, one fast and one slow, to alter circuit response sequentially. For example, rapid STAR-triggered activation and slow CRISPRi were combined to elicit a pulse of output signals upon induction^107^.

### Multi-level regulation reduces genetic footprints

Exerting multi-level regulation unleashes circuits’ capabilities for assimilating inputs into bespoke output patterns. In this multi-level architecture, different regulatory systems play unique roles in attaining the desired function with the smallest genetic footprints: the fewest regulatory parts and transcriptional layers. The regulatory mechanisms at different levels also tend to operate independently, offering inherent orthogonality and thus lowering the requirements for the number of orthogonal parts in a single regulatory level. Muldoon et al.^94^ demonstrated these benefits by creating AND, IMPLY, NAND, and NIMPLY logic gates using only one split intein and one zinc-finger protein (ZFP) in the mammalian system. They extended the framework to implement two-input-two-output circuits and analog signal processing predictably. Likewise, Doshi et al.^108^ devised a single-layer six-input disjunctive normal form-like mammalian circuit comprising AND, OR, and NOT logic gates, accomplished via the cooperation of TF, intron alternative splicing, and miRNA (Fig. 3c). More recently, the transcriptional autoregulation and post-translational competitive interactions were also incorporated for achieving multistability in minimal circuitry, generating up to seven stable cell states using three ZFPs and one chemical-inducible dimerization domain^13^ (Fig. 3d).

Another example is CRISPR-based logic circuits. The CRISPRi-based NOT and NOR gates have been interconnected into two-input logic gates, among which the AND and NAND gates were built from six sgRNAs^109^. However, these gates can be constructed using fewer sgRNAs by incorporating crRNA-tracrRNA interaction with CRISPRa^110^ or protein-splicing with CRISPRi systems^69^. The multipartite assembly of gRNAs and synthetic RNAs^111^ further scaled up these computations. Moreover, the IMPLY logic has not been demonstrated in the layered design but in a multi-level design combining CRISPRi with the asRNA system^71^. Reducing the number of simultaneously expressed sgRNAs is beneficial for maintaining high dynamic ranges of CRISPRi-based regulation^112^, as they share finite dCas9 resources, which are tightly controlled to avoid the toxic effects on host cells.

### Multi-level regulation implements genetic controllers

The performance of genetic circuits is substantially affected by their working environment (context), which is a slew of genetic, cellular, and extracellular conditions interacting with the circuits. Assembling different genetic parts changes the local DNA sequences (intragenic contexts) and potentially affects the original activities of each part or gives rise to new genetic parts, disrupting normal functions. The intergenic contexts (e.g., the location, orientation, and order) of genetic circuits on plasmids or genomes also alter circuit responses^113^. Most genetic circuits share limited cellular resources (e.g., the transcription, translation, and degradation machinery) for performing functions, thus being amenable to resource availability and variations, intrinsic cellular noises, and cell types^114^. The extracellular conditions (e.g., temperature, pH, and growth medium) also alter circuit performance by perturbing host cell states and genetic part activities. There has been a growing awareness of the context effects, and a set of genetic controllers have been developed to contend with these effects (systematically reviewed in^115^). These genetic controllers generally employ negative feedback (NF) or incoherent feedforward loop (iFFL) topologies, containing TF-driven activation paths and repression paths that could be attained via RNA or protein interactions.

A landmark of the NF controller is the synthetic implementation of integral controller, a strategy natural circuits adopt for maintaining homeostasis, requiring a pair of stable molecule species to annihilate each other in the 1:1 stoichiometric ratio. This interaction was obtained by sigma/anti-sigma sequestration^116^ or split intein^95^ systems, resulting in robust perfect adaptation to environmental perturbations (Fig. 3e). Likewise, quasi-integral controllers were enabled by asRNA/mRNA interactions to impart adaptations to fluctuations in ribosome availability^117^ and various disturbances^118^. The asRNA- and TF-based NFs were coupled into a layered feedback controller^119^, affording improved robustness and faster resettling to attenuate chemical, temperature, and nutrient perturbations. Furthermore, the iFFL controllers also adopted post-transcriptional repression mediated by miRNA^120^ or endonuclease^73^ for offsetting the effects of disturbances on output signals. These iFFL controllers have been demonstrated to buffer gene expression against noise and external perturbations, buffer gene dosage (or copy number) variation, and mitigate gene expression burden (Fig. 3f).

### Multi-level regulation facilitates real-world applications

Synthetic biology has revolutionized biosensing, bioproduction, and biotherapeutics, beginning to deliver real-world products for addressing global needs. The multi-level regulatory circuits expedite the applications of these cellular workhorses by improving their functionality, stability, and safety.

Biosensing functions are essential for developing whole-cell sensors in disease diagnosis, contaminant monitoring, and hazard detection. They are also crucial for implementing controllers and increasing target specificity in “smart” bioproduction and biotherapeutics. Although diverse regulatory mechanisms have shown sensing functions, their dose-response profiles must be fine-tuned to match the practical needs, which could be attained by multi-level regulation without painstakingly reengineering the sensors. For example, riboswitches usually suffer from low dynamic ranges, which could be magnified by introducing TF^121^, plasmid copy number (PCN) control^50^, and recombinases^122^. In addition, different regulatory modalities add diversified functions to the sensing circuits, like integrating signals from multiple sensors^123^ and signal recording^124^.

In many application scenarios, the engineered cells were expected to execute burdensome or toxic functions and operate over long periods or in outside-the-lab settings, which causes genetic instability, that is, the accumulation of mutations disrupting desired function. To address this issue, a terminal differentiation strategy^51^ integrating RNAP, recombinase, PCN control, and protein splicing was recently developed in *E. coli* (Fig. 3g). During the differentiation process, the recombinase excises the expression cassette of the intein-split DNA replication protein, ceasing the replication of the control plasmid and resulting in the loss of antibiotic resistance. Meanwhile, the T7 RNAP expression is elicited to activate the gene-of-interest (GOI) expression. Therefore, the progenitor cells could proliferate but not express GOI, whereas the differentiated cells are the opposite. The terminal differentiation increased bacterial workhorses’ shelf-stability and long-term performance and empowered the continuous production of toxic protein Dnase I.

Furthermore, incorporating RNA- and protein-level regulation facilitates the development of RNA therapeutics. Compared with their DNA counterparts, RNA-delivered circuits evoke transient gene expression and exhibit reduced risks of insertional mutagenesis, immunogenicity, and epigenetic silencing, holding great promise for treating countless diseases. A clear example of this is the mRNA vaccines developed against SARS-CoV-2. Therefore, some research groups explored post-transcriptional regulation tools that can be encoded in and act on mammalian RNAs, such as miRNA, RNA binding proteins (RBPs), and endonuclease. Using RBPs as a bridge, protein cleavage^125^ and degradation^126^ were introduced to expand the operational landscape of RNA-delivered circuits. These circuits could sense endogenous miRNA and protease biomarkers^68,125^ or external chemicals^126^ for developing cell-type-specific and spatiotemporally controllable RNA therapeutics with better safety profiles.

## Perspective and future developments

Synthetic signal processing circuits have flourished in the past decade. Endeavors to implement these circuits benefit from the emerging novel regulatory modalities and advanced tools for computer-aided design. These achievements also counted on efforts to optimize the design and improve the processing capabilities of single regulatory modalities. Indeed single-level circuit permits predictable circuit design by matching the input-output profiles in transcriptional circuits and the composition of parts operating at the same timescale, which is especially crucial for maintaining fast kinetics of RNA- and protein-only circuits. However, a marriage of different regulatory modalities will enact a multi-level platform where each modality finds its niche and cooperates to execute complex functions. The multi-level circuits divide the desired tasks into separate modules, allowing each part to harness its strength. We thus envision that the multi-level regulation will tremendously augment the current circuit design paradigm.

Despite its great promise, designing such multi-level circuits requires careful consideration to circumvent practical pitfalls. Introducing RNA- and protein-level regulation may cause detrimental effects on circuit behavior by modifying RNA and protein sequences and disrupting their structures, which also raised difficulties in predicting the circuit’s performance from the characterization data of each part. Opportunities to tackle these issues are provided by a wealth of computational tools for de novo RNA and protein structure design and sequence-to-function prediction, empowered by mechanistic models and machine-learning algorithms. An alternative strategy is to curate a set of compatible and composable parts. For example, the asRNA-mediated control will not modify target RNA sequences and could be readily co-opted into current TF-based frameworks^127^. Moreover, the prototyping and optimization of the circuits could be expedited with the aid of active learning algorithms^128^ and high-throughput screening workflows like transposon-based approaches^65,91^ and massive parallel reporter assays. Screening the time-varying dynamics of circuit variants is also rendered feasible by coupling parallelized microfluidics and time-lapse fluorescence microscopy^129^. Finally, the design automation of multi-level circuits will benefit from precisely quantifying gene expression at different levels by RNA-seq^114,130^ and establishing large libraries of standardized, modular parts and devices with multiple inputs and outputs.

Advances in several facets will also benefit genetic circuit design and application. First, improvements in our capabilities of de novo designing biological parts, especially RNA and protein elements, will yield artificial parts that function well at as low concentrations as several copies per cell, reducing the resource consumption of synthetic circuits. Next, regulatory tools and genetic circuit design principles need to be developed in clinically or industrially important organisms and evaluated in contexts of application scenarios. The regulatory modalities bypassing host machinery, such as ribozymes, CRISPRi, and inteins, will be valuable for developing portable circuit design automation platforms and genetic controllers for predictable and robust functions in non-model organisms. Finally, understanding, accommodating, and harnessing the biological properties of living systems will increase synthetic circuits’ complexity to the levels of natural circuits. For example, the crosstalk among genetic parts has been regarded as trouble to overcome; however, promiscuity is prevalent in natural biological interactions and can be harnessed to design complex “many-to-many” circuit networks^131^. Natural signal processing leverages the cooperation of gene regulation, metabolic regulation, and signal transmission. The latter two remain less explored in genetic circuit design. On the one hand, merging metabolic and gene regulation will yield complex circuits that process signals by enzyme-catalyzed biochemical reactions and transduce signals to bespoke outputs by metabolite-responsive regulatory parts^132,133^. On the other hand, intercellular signal transmission enables multicellular distributed computation^42,134^, where computation tasks are distributed among different cells to reduce circuit complexity in single cells and exploit concurrency^135^. Integrating signal transmission with gene regulation will point to unprecedentedly complex signal processing circuits, requiring novel intercellular communication modules like cell-to-cell RNA delivery^136^ and automated workflows^137^ to design synthetic cellular populations.

As synthetic biology permeates society, the knowledge from different regulatory modalities, fields, and disciplines must converge to customize signal processing circuits to address real-world challenges. The advancements in cellular signal processing will also innovate and accelerate the development of synthetic cell consortia, cell-free systems, and biotic/abiotic interfaces, tremendously expanding the potential application space of synthetic biology.
